# Quality of guidelines on the management of diabetes in pregnancy: a systematic review

**DOI:** 10.1186/1471-2393-12-58

**Published:** 2012-06-28

**Authors:** Marjolein JE Greuter, Nathalie MA van Emmerik, Maurice GAJ Wouters, Maurits W van Tulder

**Affiliations:** 1 Department of Health Sciences & EMGO + Institute for Health and Care Research, Faculty of Earth and Life Sciences, VU University, De Boelelaan 1085, 1081, HV Amsterdam, The Netherlands; 2 Department of Obstetrics and Gynaecology, VU University Medical Center, Amsterdam, the Netherlands

**Keywords:** Clinical guidelines, Diabetes mellitus, Gestational diabetes, Pregnancy

## Abstract

**Background:**

Diabetes during pregnancy can lead to severe risks for both mother and fetus when it is not managed properly. The use of rigorously developed guidelines with a robust implementation process can have a positive influence on the management of diabetes during pregnancy. This study aims to compare recommendations and assess the quality of clinical guidelines on gestational diabetes mellitus (GDM) and pre-existing diabetes mellitus during pregnancy.

**Methods:**

Guidelines were selected by searching PubMed, the Guideline Clearing House and Google. All guidelines developed since 2000 on diabetes during pregnancy in English or Dutch were considered. Recommendations of the guidelines were compared. Furthermore, the quality was assessed by two authors independently, using the AGREE instrument.

**Results:**

Eight guidelines were included. According to the AGREE instrument, the quality of most guidelines was low. The domains editorial independence, stakeholder involvement and rigour of development had the lowest scores. Recommendations were mainly comparable on glycemic control, preconceptional counseling and prenatal care and labour. Differences between recommendations were found for screening on GDM and induction of labour.

**Conclusions:**

The quality of most guidelines concerning the management of diabetes during pregnancy needs to be improved. A more systematic approach in the development of these guidelines, more attention for updating procedures and piloting of the guidelines and involvement of target users and patients is recommended.

## Background

Diabetes during pregnancy can lead to serious risks for both mother and fetus [1]. The management of diabetes differs between women with gestational diabetes mellitus (GDM) and pregnant women with pre-existing diabetes mellitus type 1 (DM1) or type 2 (DM2).

GDM develops often towards the end of the second trimester [1]. Maternal risk factors for GDM are: a BMI above 30 kg/m², history of unexplained intrauterine fetal death, previous GDM or a macrosomic baby, family history of DM, polycystic ovarian syndrome and ethnicity. Especially women of South Asian, Black Caribbean and Middle Eastern origin have an increased risk of GDM [2-4]. After delivery, GDM usually resolves. However, both mother and child have an increased risk of developing DM2 later in life.

In the Netherlands, the prevalence of GDM is estimated between 1-15%, depending on the study population [5]. The highest prevalence is found in non-Dutch women. Because of migration and the increasing prevalence of obesity, it is likely that the number of pregnancies complicated by GDM will increase.

DM1 and DM 2 affect 1% of pregnancies, but this is probably an underestimation [6]. Due to the increasing prevalence of DM2 caused by obesity in a younger age group, it is assumed that the prevalence of pregnancies complicated by DM2 will increase as well.

The possible complications due to diabetes during pregnancy are severe. The mother has an increased risk of pre-eclampsia, infections, ketoacidosis, hypoglycemia and micro-vascular diseases such as retinopathy, nephropathy and neuropathy [7]. In addition, there is an increased risk for miscarriage, still birth, congenital defects and neonatal morbidity and death [8]. For congenital defects, a dose-response relation is found: the poorer the periconceptional blood glucose control, the greater is the risk on congenital defects [9]. Another major complication is macrosomia, which is a risk factor for instrumental delivery, caesarean section, shoulder dystocia during delivery and neonatal hypoglycemia directly after birth [10].

There is strong evidence that proper management of GDM and pre-existing DM during pregnancy leads to better health outcomes for both mother and child [1,11]. To ensure proper management, several guidelines from different countries, institutes and organisations concerning the management of diabetes during pregnancy have been published. In general, guidelines are important instruments for improving the quality of health care. They should be based on the best available evidence and should also take patient preferences and clinical experience into account [12]. Furthermore, an effective strategy for the implementation of recommendations is an important next step for realising proper management.

So far, the quality of guidelines concerning diabetes in pregnancy has not been established. Therefore, the aim of this study was to assess the quality of guidelines on diabetes during pregnancy by an internationally acknowledged instrument. In addition, the recommendations for the management of diabetes during pregnancy were compared.

## Methods

### Selection of guidelines

To identify relevant clinical guidelines, the database of PubMed (Medline) was searched up to October 2011 using the following terms: *diabetes, gestational diabetes* and *pregnancy* and limited by ‘clinical guidelines’*.* Because guidelines are often not published in medical journals, the search was extended to the Internet by screening the Guideline Clearing House and Google. When searching these databases, the following terms were used: *clinical guidelines, diabetes, gestational diabetes* and *pregnancy.* The authors checked the links in Google until saturation was reached.

To be included in this study, guidelines had to meet the following criteria:

1) the patient group consisted of pregnant women with GDM or pre-existing DM, 2) the guideline addressed the management of GDM or pre-existing DM during pregnancy, 3) full text was available on the internet, 4) the guideline concerned Western populations and 5) the guideline was available in the English or Dutch language.

Guidelines were excluded if they focused on the management of diabetes mellitus in general and did not include pregnant subjects. Furthermore, reports that provided reviews on guidelines but did not contain specific recommendations were excluded, as well as guidelines that were developed before 2000 or that had not been updated in the last 10 years.

### Quality assessment

Two reviewers independently assessed the quality of the guidelines by using the English version of the AGREE instrument [12]. The AGREE instrument consists of 23 items in six domains (Table 1), which includes: 1) scope and purpose of the guideline, 2) stakeholder involvement, 3) rigour of development, 4) clarity and presentation, 5) applicability and 6) editorial independence.

**Table 1:** Domains and Items of the AGREE Instrument

**AGREE Domain**	**AGREE Item**
Scope and purpose	The overall objective of the guideline is specifically described
	The clinical question covered by the guideline is specifically described
	The patients to whom the guideline is meant to apply are specifically described
Stakeholder involvement	The guideline development group includes individuals from all relevant professional groups
	The patients’ view and preferences have been sought
	The target users of the guideline are clearly defined
	The guideline has been piloted among target users
Rigour of development	Systematic methods were used to search for evidence
	The criteria for selection of the evidence are clearly described
	The methods used for formulating recommendations are clearly described
	The health benefits, side effects and risks have been considered in formulating the recommendations
	There is an explicit link between recommendations and supporting evidence
	The guideline has been externally reviewed by experts prior to its publication
	A procedure for updating the guideline is provided
Clarity and presentation	The recommendations are specific and unambiguous
	The different options for the management of the condition are clearly presented
	Key recommendations are easily identifiable
	The guideline is supported with tools for application
Applicability	The potential organizational barriers in applying the recommendations have been discussed
	The potential cost implications of applying the recommendations have been considered
	The guideline presents a key review criteria for monitoring and/or audit purposes
Editorial independence	The guideline is editorially independent from the funding body
	Conflicts of interest of guideline development members have been recorded

Each item was assessed on a 4-point scale: 1 = strongly disagree; 2 = disagree; 3 = agree and 4 = strongly agree. The additional information in the AGREE guide was used in order to clarify and correctly interpret each item. Differences between the scores on positive and negative assessment (e.g. scoring 1 or 2 *vs.* 3 or 4) of the two reviewers were resolved in a consensus meeting. Finally, domain scores were calculated by dividing the differences between the obtained score and the minimum possible score by the difference between the maximum possible score and the minimum possible score. In line with similar studies, we defined scores of 50% or less as low quality [13,14].

### Comparison of guidelines

Two reviewers individually summarized four guidelines each. Focus of the summaries was on recommendations. Each summary was checked by the other reviewer on clarity and completeness. One reviewer independently compared the recommendations of all summaries and the other reviewer examined if these comparisons were correct.

## Results

Four guidelines were identified in PubMed, four in the Guideline Clearing House and seven in Google. Guidelines were excluded for different reasons; the guideline of the International Diabetes Center [15] was excluded because this guideline was similar to the guideline of the American Diabetes Association [16,17]. Other guidelines were excluded because they did not concern Western populations [18], did not focus on diabetes in pregnancy [19,20] or because of publication date [21,22].

Eight guidelines were included in this study, namely:

1) *American College of Obstetricians and Gynecologists (ACOG)*[23]

2) *American Diabetes Association (ADA), GDM*[16]*and pre-existing DM*[17]

3) *Australasian Diabetes in Pregnancy Society (ADIPS), GDM*[24]*and pre-existing DM*[25]

4) *British Columbia Reproductive Care Program (BCRCP) *[26,27]

5) *Colorado Clinical Guidelines Collaborative (CCGC)*[28]

6) *Dutch Society of Obstetrics and Gynaecology (DSOG)*[29]

7) *International Diabetes Federation (IDF) *[30]

8) *National Institute for Clinical Excellence (NICE) *[31]

### Quality assessment

There was a high level of agreement between the two independent reviewers. In general, the quality of most guidelines was questionable when using the AGREE instrument. The domains with the highest scores were applicability, clarity and presentation and scope and purpose. The domains editorial independence, stakeholder involvement and rigour of development had the lowest scores. The domain scores of each guideline are shown in Table 2.

**Table 2:** Domain Scores of the guidelines (percentages)

	**Guideline**							
**Agree Domain**	**1**	**2**	**3**	**4**	**5**	**6**	**7**	**8**
Scope and purpose	67	56	39	33	28	61	67	94
Stakeholder involvement	17	8	42	4	14	21	8	100
Rigour of development	48	29	38	12	31	31	38	93
Clarity and presentation	67	71	75	50	83	63	71	100
Applicability	44	56	56	33	39	56	94	89
Editorial independence	0	0	42	0	0	8	50	50

1, American College of Obstetricians and Gynecologists, 2005. 2, American Diabetes Association, 2003 & 2008. 3, Australasian Diabetes in Pregnancy Society, 2002 & 2005. 4, British Columbia Reproductive Care Program, 2001. 5, Colorado Clinical Guidelines Collaborative, 2006. 6, Dutch Society of Obstetrics and Gynaecology, 2010. 7, International Diabetes Federation, 2009. 8, National Institute for Clinical Excellence, 2008.

The majority of the guidelines described their scope and purpose quite specific. Four guidelines adequately described both objectives and patient groups [16,17,29-31]. In the other guidelines description of objectives was incomplete [23-28]. Moreover, specific clinical questions were only provided by the NICE guideline [31].

Regarding stakeholder involvement, seven guidelines scored below 50%. Patients’ view and preferences were often not included in the development of the guideline. Moreover, the guidelines had not been piloted among target users, with the exception of the NICE guideline [31].

Seven guidelines had a low score on rigour of development. This was mainly because most guidelines did not describe explicitly how they had identified, selected and summarized the available evidence. Also information on updating was not reported adequately in most guidelines; only the NICE guideline [31] provided a procedure for updating.

In contrast, except for the guideline from the BCRCP [26,27], all guidelines provided information on health benefits, side effects or adverse effects. In addition, most guidelines provided an explicit link between recommendations and evidence by references or a summary of the evidence. Only the CCGC guideline [28] did not provide adequate information on the relation between evidence and recommendation.

Regarding clarity and presentation, seven guidelines scored over 50%. Key recommendations were easily identifiable and different options for management were given. It should be noted that only the CCGC guideline [28] and NICE guideline [31] were supported with tools for application, such as a quick reference guide.

On applicability, five guidelines scored over 50%. All guidelines presented key review criteria for monitoring and/or audit purposes. Considerations on costs or additional resources were described by four guidelines [16,17,24,25,30,31]. The guidelines of the DSOG [29], IDF [30] and NICE [31] also provided information on potential organizational barriers.

Regarding editorial independence, all eight guidelines scored at or below 50%. Five guidelines did not report anything on both independency of funding body and conflicts of interest [16,17,23-25,28,29]. The other three guidelines only fulfilled one of the two items [26,27,30,31].

### Comparison of recommendations on management of diabetes during pregnancy

The recommendations in the guidelines can be divided into four domains, namely screening for GDM, glycemic control, prenatal care and labour and preconception counselling for women with DM1 or 2. The similarities and differences between the different guidelines on each domain will be discussed.

The first domain is screening for GDM. The recommendations in this domain were inconsistent. Three guidelines recommended that all pregnant women should be screened [24,25,28,30]. On the other hand, four guidelines recommended that only women with risk factors should be screened [16,17,26,27,29,31]. However, some guidelines refined their recommendation. The ADIPS guideline [24,25] stated that selective screening should be used when resources are limited. Furthermore, the IDF guideline [30] stated that when the effectiveness of selective screening is shown, this should be recommended.

Not all guidelines were explicit about what screening criteria to use and at what time the screening should take place. Regarding the screening criteria, two guidelines stated that the Coustan & Carpenter criteria should be used [26-28] and one guideline [24,25] recommended modified WHO-criteria. Concerning the time of screening, the BCRCP guideline [26,27] stated that it is not important to screen early while the CCGC guideline [28] distinguished between women with and without risk factors. Women with risk factors should be screened as early in pregnancy as possible. For women without risk factors, early screening is not necessary.

The second domain is glycemic control. All guidelines made similar recommendations such as, among others;

Use a multidisciplinary approach that is adjusted to the individual.

Primary strategy is focused on nutrition and physical activity.

When glucose levels remain too high, medication therapy should be started.

Insulin is the primary choice; the use of hypoglycaemic agents is discouraged.

Blood glucose should be self-monitored regularly and the importance of maintaining normal glucose levels should be emphasized.

The third domain is prenatal care and labour. Two recommendations were similar for most guidelines, namely; 1) extra fetal surveillance is not necessary unless there are complications and 2) during labour, it is important to maintain normal glucose values. Therefore, glucose values should be monitored regularly.

Some guidelines made additional recommendations. For example, the ADIPS guideline [24,25] stated that after delivery, women with pre-existing DM should be monitored closely to find a new balance. Furthermore, some guidelines [16,17,23,29-31] stated that during prenatal care there should be searched for possible complications.

However, there was one important difference between the guidelines. Four guidelines recommended that delivery should not take place before full term unless there are complications [23-28]. In contrast to this, three guidelines stated that delivery should be induced after approximately 38 weeks of gestation [16,17,29,31]. The IDF guideline [30] did not make recommendations on this topic.

The last domain is preconception counselling for women with pre-existing DM. The recommendations in this domain were similar for most guidelines. The first recommendation was that all women with DM1 or 2 in reproductive age should receive counselling. This counselling should include information about the risks in pregnancies complicated by diabetes. Also the importance of normal glucose values before conception should be stressed [16,17,23-31]. Other recommendations in this domain discussed the use of medication. Medication that is used before conception, for example hypoglycaemic agents, should be evaluated to determine if it is safe during pregnancy [16,17,23-31]. In addition, it must be checked if there are any complications, such as retinopathy, present before conception [16,17,23,29].

## Discussion

The use of clinical guidelines is likely to have a positive influence on the management of diabetes during pregnancy [1,11]. This article provides an overview of the quality and content of clinical guidelines regarding the management of diabetes during pregnancy. We reviewed eight guidelines from six different countries. This study shows that the overall quality of these guidelines was low when using the AGREE instrument. Only two guidelines were found to be of moderate [30] or high quality [31]. In general, the recommendations in several domains were similar except for screening for GDM and induction for delivery at term.

It is important that guidelines are of high quality. According to the AGREE instrument, most guidelines inadequately reported on editorial independence, stakeholder involvement and rigour of development. Especially the low score on rigour of development is concerning, because explicit descriptions of how the available evidence was identified and selected is essential for the development of valid and reliable evidence-based recommendations.

Also descriptions of the updating procedures of the guidelines were poor, although it is important to keep the recommendations based on the best available evidence. Regarding stakeholder involvement, it might be important that views and preferences of patients are taken under consideration and that guidelines are piloted among target groups. This may increase success of implementation and thereby improve the management of diabetes during pregnancy.

The low quality of most guidelines could be explained by the fact that the AGREE instrument was not used by the committees that were developing or updating the guidelines. Only the committee of the NICE guideline [31] used an instrument to asses the quality which is part of their rigorous, standardized procedures. The use of the AGREE instrument already showed to improve the quality of other guidelines, such as guidelines on the management of low back pain [14,32].

It should be noted that the score on the AGREE instrument does not only depend on the methodological quality of the guideline, but also on the quality of reporting. It is possible that guidelines of high methodological quality score low on the AGREE instrument due to poor reporting. One should bear this in mind while interpreting the results of the current study. However, most guidelines were published years after the AGREE instrument was first published. One would assume that guideline developers are aware of the most up to date discussions and literature about quality of developing and quality of reporting guidelines.

Although there is some debate about using the AGREE instrument for assessing the quality of guidelines and about a 50% cut-off value, other papers on back pain and acute gastroenteritis have used this approach before [13,14]. Obviously, if the quality criteria were stricter, then even fewer guidelines would be considered of good quality, as shown in Table 2.

Regarding the recommendations, there were some discrepancies between guidelines. These differences might be a result of lack of evidence or weak associations. For example, recommendations regarding screening on GDM in the guidelines diverged as was also found in other studies [33,34]. This diversity might be caused by equivocal evidence on this topic in the period in which most guidelines were developed [35]. Also consensus usually does not warrant similar recommendations because not only the available evidence but also other aspects such as costs, applicability, constitution of the guideline committee and ethical considerations influence recommendations.

Nonetheless, more variety between recommendations was expected because of the variety in health care systems, culture in various countries and the differences in membership of the guideline committees. Therefore, current scientific evidence on the management of diabetes seems to be appropriate to generalize conclusions to these different groups. Moreover, previous studies also showed that international guidelines were consistent in most of their recommendations, especially on preconceptive care in women with diabetes [36].

The similarities between the guidelines included in this study could partly be explained by the fact that they all have been developed between 2001 and 2010. Moreover, the recommendations of the ADA guideline [16,17] and the CCGC guideline [28] were based mainly on the same literature. Also some references of the IDF guideline [30] were similar to those of the ADA [16,17] and the NICE guideline [31]. In addition, the recommendations of the guideline of the DSOG [29] were partially based on the NICE guideline [31].

## Conclusions

The quality of most guidelines on the management of diabetes during pregnancy can be improved. A more systematic approach in the development and reporting of these guidelines is recommended. Extra attention for updating procedures is advised. Also involvement of different health care professionals and patients in the development and evaluation of guidelines is necessary to obtain successful implementation. In addition, more attention for piloting of guidelines is recommended. In order to obtain and evaluate the improvements as described above, the AGREE instrument can be a helpful tool.

However, guidelines of good quality do not ensure good quality of management in daily practice. Therefore, further research is important to develop efficient implementation strategies to increase appropriate uptake of guideline recommendations by health care professionals.

## Competing interests

The authors declare that they have no competing interests.

## Authors’ contributions

All authors have made substantial contributions and gave final approval of the conceptions, drafting, and final version of this manuscript. MG and NE contributed equally to the data collection, review of the guidelines, interpretation of the data and the writing of this manuscript. MT provided the idea for this project, supervised the procedures in the study and reviewed the drafts and final version of this manuscript in concert with MW. All authors have seen and approved the final version of the manuscript.

## Details of ethics approval

Ethics approval was not needed for this systematic literature review.

### Funding

No funding was received.

## Pre-publication history

The pre-publication history for this paper can be accessed here:

http://www.biomedcentral.com/1471-2393/12/58/prepub
